# Metabolism of Phenolics of *Tetrastigma hemsleyanum* Roots under In Vitro Digestion and Colonic Fermentation as Well as Their In Vivo Antioxidant Activity in Rats

**DOI:** 10.3390/foods10092123

**Published:** 2021-09-08

**Authors:** Yong Sun, Fanghua Guo, Xin Peng, Kejun Cheng, Lu Xiao, Hua Zhang, Hongyan Li, Li Jiang, Zeyuan Deng

**Affiliations:** 1 State Key Laboratory of Food Science and Technology, Nanchang University, Nanchang 330047, China; yongsun@ncu.edu.cn (Y.S.); gfh1376234247@outlook.com (F.G.); 2 College of Food Science and Technology, Nanchang University, Nanchang 330047, China; xl2691915147@163.com; 3 Ningbo Research Institute, Zhejiang University, Ningbo 315100, China; pengx@nit.zju.edu.cn; 4 Chemical Biology Center, Lishui Institute of Agriculture and Forestry Sciences, Lishui 323000, China; chengkejun@gmail.com; 5 College of Pharmacy, Jiangxi University of Traditional Chinese Medicine, Nanchang 330004, China; sunnymay_z@hotmail.com (H.Z.); viviface@yeah.net (L.J.)

**Keywords:** *Radix Tetrastigma*, colonic fermentation, phenolics, antioxidant capacity, bioavailability

## Abstract

*Tetrastigma hemsleyanum* Diels et Gilg is a herbaceous perennial species distributed mainly in southern China. The *Tetrastigma hemsleyanum* root (THR) has been prevalently consumed as a functional tea or dietary supplement. In vitro digestion models, including colonic fermentation, were built to evaluate the release and stability of THR phenolics with the method of HPLC-QqQ-MS/MS and UPLC-Qtof-MS/MS. From the oral cavity, the contents of total phenolic and flavonoid began to degrade. Quercetin-3-rutinoside, quercetin-3-glucoside, kaempferol-3-rutinoside, and kaempferol-3-glucoside were metabolized as major components and they were absorbed in the form of glycosides for hepatic metabolism. On the other hand, the total antioxidant capacity (T-AOC), superoxide dismutase (SOD), glutathione peroxidase (GSH-Px) activity, and glutathione (GSH) content were significantly increased, while malondialdehyde (MDA) content was decreased in plasma and tissues of rats treated with THR extract in the oxidative stress model. These results indicated that the THR extract is a good antioxidant substance and has good bioavailability, which can effectively prevent some chronic diseases caused by oxidative stress. It also provides a basis for the effectiveness of THR as a traditional functional food.

## 1. Introduction

*Tetrastigma hemsleyanum* Diels et Gilg, a herbaceous perennial species, is distributed mainly in southern China. The *Tetrastigma hemsleyanum* root (THR, called “Sanyeqing” in Chinese) is one of the functional foods commonly used in China. As an edible plant, THR has been prevalently consumed as a dietary supplement, nutrient, or health tea for its health benefits. Meanwhile, the specific bioactive properties of THR such as immunoregulatory, antioxidant, anti-inflammatory, antipyretic, analgesic, and antiproliferactive capacity have been widely reported. It has also been applied to treat high fever, infantile febrile convulsion, pneumonia, asthma, hepatitis, rheumatism, menstrual disorders, sore throat, and scrofula [1,2]. In our previous publication, abundant flavonoids and phenolic acids were found in THR, which may be responsible for their bioactivity above. Moreover, the phenolics extract from THR showed strong antioxidant activities in several in vitro assay systems (DPPH, FRAP, and ABTS) [3]. Although the phenolics extract from THR has strong in vitro antioxidant capacity, few studies have reported its in vivo antioxidant effects.

The overproduction of reactive oxygen/nitrogen species (ROS/RNS), e.g., superoxide (O_2_^−^), hydrogen peroxide (H_2_O_2_), hydroxyl radical (OH), singlet oxygen (O_2_), nitric oxide (NO·), peroxynitrite (ONOO^−^), and nitrogen dioxide (NO_2_), leads to an imbalance between the pro-oxidant and the antioxidant, which is the main cause of oxidative stress [4,5]. Under normal physiological conditions, these active substances are not necessarily a threat to the body. However, when they are unable to be cleared by the body to a certain degree, the risk of diseases increases, e.g., inflammation, cancer, type 2 diabetes mellitus (T2DM), and atherosclerosis [5,6]. To combat the oxidative stress, a defense system was established, which refers to various antioxidant enzymes, including superoxide dismutase (SOD), gluthatione peroxidase (GSH-Px) and catalase (CAT) [7]. On the other hand, oxidative stress induces lipid peroxidation [8], and malondialdehyde (MDA), which is a peroxidation product of polyunsaturated fatty acids, was detected as the index of lipid peroxidation [9]. There is lack of information on how these phenolics extracts from THR affect the in vivo antioxidant enzymes and MDA.

Dietary polyphenols have received increasing attention in recent years, due to their potential antioxidant properties [10]. Generally, the in vivo bioactivity of phenolics (including antioxidant activity) is significantly distinct from the in vitro data; therefore, the phenolic content in food does not reflect its absorption and metabolism in the human body [11]. For example, the first pass effect of xenobiotics, as well as low phenolic absorbability, leads to an extremely low phenolic concentration in systematic circulation [12]. Meanwhile, the structure modification during metabolism in the body has a considerable influence on the bioactivity as well. In contrast, the liberation of phenolics from matrices during digestion and colonic fermentation potentially promote the concentration of phenolics in plasma. In parallel, the structure alteration yielded by pH, enzymes, and gut microbials also contribute to the bioavailability and bioactivity of phenolics [13]. Mosele et al. [14] found that intestinal microbes were beneficial for polyphenol metabolism when they studied pomegranate products (juice, pulp, and peel extract) with in vitro gastrointestinal digestion and colonic fermentation models. However, the release rate and stability of THR phenolics during the digestion process as well as the specific routine of microbial metabolism during colonic fermentation are still unknown.

In this paper, in vitro simulated gastrointestinal digestion and colonic fermentation models were built to evaluate the stability and catabolism of *Tetrastigma hemsleyanum* phenolics combined with HPLC-QqQ-MS/MS and UPLC-Qtof-MS/MS methods, which have been used to identify metabolites from animals or humans. Furthermore, after oral administration of THR extract to rats, the regulating effects of these phenolics and/or their metabolites on the antioxidant enzyme activities, the total non-enzymatic antioxidant capacity, and lipid peroxidation in plasma and different tissues of rats were also investigated.

## 2. Materials and Methods

### 2.1. Materials and Reagents

Fresh *Tetrastigma hemsleyanum* roots were purchased from Jiangxi Shangrao Red Sun Agricultural Development Co., Ltd. (Nanchang, China). Pepsin, bile salts, and pancreatin were obtained from Aladdin Co. Ltd. (Shanghai, China). Human salivary a-amylase, DPPH, and DMSO were purchased from Sigma-Aldrich Co. (St. Louis, MO, USA). Methanol, acetonitrile, formic acid, and other solvents and chemical agents were obtained from Merck (Darmstadt, Germany). Water was purified in-house by a Milli-Q system (Bedford, MA, USA). The superoxide dismutase (SOD), glutathione peroxidase (GSH-Px), total antioxidant capacity (T-AOC), reduced glutathione (GSH), and malondialdehyde (MDA) kits were purchased from Nanjing Jiancheng Bioengineering Company (Nanjing, China).

### 2.2. Extract Preparation

THR samples were dried until constant weight by an electro-thermostatic blast oven (Senxin, Shanghai, China) and ground into fine powder (through a 200 mesh sieve). The powder (1.0 kg) was extracted with 20 L 80% methanol (*v*/*v*) for 45 min at 50 °C by ultra-sonication (Shumei Instrument Factory, Kunshan, China). The suspension was centrifuged at 3000× *g* for 10 min (Thermo Scientific, Waltham, MA, USA), and was evaporated at 50 °C using a rotary vacuum evaporator (Eyela N-100, Miyagi, Japan). About 20% of its original volume remained, which was thoroughly freeze-dried (SIM International Group Co. Ltd., San Francisco, CA, USA). The dried THR extract was stored at −80 °C before use.

### 2.3. In Vitro Gastrointestinal Digestion

The in vitro digestion model was slightly adapted from Minekus et al. [15] and McClements et al. [16]. Three stages including in vitro oral, gastric, and intestinal digestion were carried out to revivify the physical and chemical effects of THR extract in the human body. All digestion processes were placed into a thermostatic water-bath shaker (37 °C, 400 rev/min), as well as away from light. Gastric and intestinal stages were air-free under nitrogen.

#### 2.3.1. Oral Digestion

The simulated salivary fluid (SSF) consisted of human salivary a-amylase (75 U/mL), CaCl_2_·2H_2_O (0.75 mM) and HBSS solution (10×) to 1× in the final stage. The SSF mixture was pre-warmed for 2 min at 30 °C. The freeze-dried THR extract was weighed (1 g) and mixed with 10 mL SSF in 50 mL polyethylene tube to perform the digestion for 5 min.

#### 2.3.2. Gastric Digestion

After oral digestion, 6 mL simulated gastric fluid (SGF) was added to the 10 mL extract-SSF mixture, and shaken for 30 min in the 37 °C water bath. To be specific, the SGF was phosphate buffer, which was adjusted to pH 2 by hydrochloride acid (1 M), and contained porcine pepsin (2000 U/mL) and CaCl_2_·2H_2_O (0.15 mM). Prior to seal-capping, most air was blown off by nitrogen.

#### 2.3.3. Intestinal Digestion

After gastric digestion, the gastric samples-chime was neutralized to pH 5.8 by dicarbonate (2 M), then mixed with simulated intestinal fluid (SIF), which consisted of pancreatin, bile salts, and CaCl_2_·2H_2_O at a final concentration of 100 U/mL, 0.01 mM, and 0.3 mM, respectively. Then, the mixed digestive juice was adjusted to pH 8 with NaOH for digestion for 8 h. PMSF was added to terminate the reaction with a final concentration of 0.174 mg/mL.

The digestive juices of each stage were freeze-dried, including oral digestive products (THR-O), gastric digestion products (THR-G), and intestinal digestive products (THR-I). Then, all dried samples were re-dissolved with 80% methanol, which were purified by Agilent C_18_ solid phase extraction column and stored at −80 °C before analysis. All samples were taken 3 times in parallel.

### 2.4. In Vitro Colonic Fermentation

The in vitro fermentation colonic model was based on Maccaferri and Pereira-Caro with slight modification [17,18].

#### 2.4.1. Preparation of Anaerobic Medium

A total of 7.5 g bouillon culture-medium was dissolved in 250 mL of distilled water and the mixture was filtered. The filtrate was sterilized at 120 °C for 20 min, and 1.5 mg hemin and 0.25 μL vitamin K_1_ were added when the temperature of filtrate dropped to room temperature.

#### 2.4.2. Preparation of Human Intestinal Bacterial Suspension

Three healthy young female volunteers (24–40 years old, who had not taken any antibiotics or fungi within 3 months before sampling and had no intestinal or metabolic diseases) were selected to collect their fresh feces. Then, the feces were mixed with anaerobic medium under anaerobic condition to make the final concentration of the fecal suspension 5%, which was filtered with gauze to obtain the intestinal bacteria solution. The operation of stools and filtration was restricted in 10 min.

#### 2.4.3. Intestinal Flora Fermentation

Under anaerobic condition, the THR extract after in vitro gastrointestinal digestion was added to the intestinal bacteria solution at a final concentration of 0.01 g/mL, which was placed into temperature-controlled anaerobic incubator (10% CO_2_, 10% H_2_, and 80% N_2_, 37 °C) for 48 h. Samples were taken at 0, 12, 24, and 48 h and freeze-dried. All dried samples were re-dissolved with 80% methanol, which were purified by Agilent C_18_ solid phase extraction column and stored at −80 °C before analysis. All samples were taken 3 times in parallel at all time points.

### 2.5. Qualitive and Quantitative Analysis of Phenolic Compounds

#### 2.5.1. Qualitive Analysis by LC-QTOF-MS/MS

LC conditions: UPLC system (Agilent 1290 infinity series) was applied to Chromatographic separation, including a degasser, a binary pump (Bin Pump SL), an injector (HiP-ALS), a column oven (TCC SL), and a DAD detector (Agilent Technologies, Santa Clara, CA, USA). Agilent Zorbax EclipseXDB-C_18_ columns (4.6 mm × 250 mm, 5 μm) and Agilent Eclipse XDB-C18 guard columns (4.6 mm × 12.5 mm, 5 μm) were used. The mobile phase consisted of A (water containing 0.2% formic acid, *v*/*v*) and B (acetonitrile containing 0.2% formic acid, *v*/*v*). The gradient elution program was as follows: 0–10 min, 14–18% B; 10–30 min, 18–20% B; 30–35 min, 20–18% B; and 35–40 min, 18–14% B. The injection volume was 10 μL, the column temperature was 40 °C, and the flow rate was 0.5 mL/min. The DAD was set between 280 and 320 nm to obtain a real-time chromatogram with absorption peaks in the range of 190–400 nm.

MS conditions: Quadrupole time-of-flight precision mass spectrometer (Agilent 6538) equipped with an electrospray ionization source (ESI) was applied in negative mode. The full mass spectral data were obtained at *m*/*z* 50–1000 mass range. The best mass spectrometry parameters were as follows: capillary voltage 3.5 kV, injection cone voltage 35 V, desolvation gas flow (N_2_) velocity 900 L/h, injection cone flow (N_2_) velocity 50 L/h, desolvation temperature 325 °C, ion source temperature 150 °C; inlet pressure was set to 10 psi in CID mode with high purity argon gas as collision gas; the CID collision energy of polyphenol monomer was set to 5, 10, 15, 20, 25, and 30 eV, and the collision energy was set to 5, 10, 15, 20, 25, 30, 40, and 50 eV; and the cracking voltage was 120 V.

#### 2.5.2. Quantitative Analysis by LC-QqQ-MS/MS

LC conditions: UPLC system (Agilent 1290 infinity series) was applied to Chromatographic separation, including a degasser, a binary pump (Bin Pump SL), a injector (HiP-ALS), a column oven (TCC SL), and a DAD detector (Agilent Technologies, Santa Clara, CA, USA). Agilent Zorbax EclipseXDB-C_18_ columns (4.6 mm × 250 mm, 5 μm) and Agilent Eclipse XDB-C18 guard columns (4.6 mm × 12.5 mm, 5 μm) were used. The mobile phase consisted of A (water containing 0.1% formic acid, *v*/*v*) and B (methanol containing formic acid, *v*/*v*). The gradient elution program was as follows: 0–5 min, 15–20% B; 5–15 min, 20–35% B; 15–25 min, 35–40% B; 25–38 min, 40–45% B; 38–45 min, 45–60% B; 45–55 min, 60–75% B; 55–65 min, 75–100% B; and 65–70 min, 100–15% B. The injection volume was 10 μL, the column temperature was 40 °C, and the flow rate was 1 mL/min. The DAD was set between 280 and 320 nm to obtain a real-time chromatogram with absorption peaks in the range of 190–400 nm.

MS conditions: Triple series quadrupole mass spectrometer (Agilent 6430 QqQ) equipped with an electrospray ionization source (ESI) was applied in negative + MRM mode. The full mass spectral data was obtained at *m*/*z* 50–1000 mass range. The best mass spectrometry parameters were as follows: capillary voltage 4 kV, injection cone voltage 35 V, desolvation gas flow (N_2_) velocity 900 L/h, injection cone gas flow (N_2_) velocity 50 L/h, desolvation temperature 300 °C, and ion source temperature 150 °C; inlet pressure was set to 15 psi with high purity argon gas as collision gas. Quantitative ion pair, collision energy, and cracking voltage were individually optimized for each monomer.

### 2.6. In Vivo Antioxidant Activities

#### 2.6.1. Animals

Sprague–Dawley (SD) rats (male, Batch No. SCXK 2015-0003), weighing approximately 200–220 g, were purchased from Shanghai Laboratory Animal Center, Chinese Academy of Sciences (Shanghai, China). They were acclimatized in an environmentally controlled breeding room for 7 days prior to treatments. The experimental protocols were approved by the Animal Ethics Committee of Nanchang University (No. 20150829).

#### 2.6.2. Experimental Design

Forty SD rats were randomly divided into five groups (*n* = 8) as follows:

Group I, normal control (saline); Group II, model control (D-galactose (D-gal) solution, 200 mg/kg BW); Group III, positive control (Vitamin C (VC) solution, 200 mg/kg BW); Group IV, low dosage (THR extract, 200 mg/kg BW); and Group V, high dosage (THR extract, 1000 mg/kg BW).

Rats in all groups were fasted for 12 h before the treatments but with access to deionized water [7]. The doses were also chosen based on the common intakes of THR in China and there was no toxic effect on the rats in our pre-experiments (data not shown). Rats in Group I were intraperitoneally injected with physiological saline, and rats in Groups II–V were intraperitoneally injected with D-gal solution (200 mg/kg BW per day). In addition, rats in Group III, IV, and V were intragastrically administered with VC, low-, and high-dose THR extract solutions at 200, 200, and 1000 mg/kg BW per day, respectively. This process lasted 20 days.

Blood samples were collected and centrifuged at 4000× *g* for 10 min. The organs (liver, heart, and kidney) of the rats were immediately dissected out, cleaned, and weighed after plasma collected. Then, they were homogenized in 10 mM Tris-HCl buffer (pH 7.4) and centrifuged at 3000× *g* for 15 min. The supernatants were analyzed for the antioxidant activities. All the above procedures were conducted at 4 °C.

#### 2.6.3. Antioxidant Assays

For the SOD, GSH-Px, T-AOC, and GSH and MDA levels, the 96-well microplates (BD Falcon, Franklin Lakes, NJ, USA) with a multi-well plate reader (Thermo Scientific varioskan flash, Waltham, MA, USA) were used according to the method reported by Li et al. [4]. Results of the enzyme activities and T-AOC capacity were expressed as units per milliliter (U/mL) in plasma or per milligram of protein (U/mg prot) in tissues.

GSH can react with 5,5′-dithio-bis-(2-nitrobenzoic acid), which was measured at 412 nm and the results were expressed as milligram per milliliter (mg/mL) in plasma or milligram per gram of protein (mg/g prot) in tissues. MDA is a product of lipid peroxide degradation that condenses with thiobarbituric acid (TBA) to form a red product with strong absorption at 532 nm [19]. The results were expressed as nanomoles per milliliter (nmol/mL) in plasma or nanomoles per milligram of protein (nmol/mg prot) in tissues [7].

### 2.7. Statistical Analysis

Results were expressed as means ± SD. All data were analyzed by the SPSS statistical software, version 19.0 (SPSS Inc., Chicago, IL, USA), which was carried out using one-way analysis of variance (ANOVA) followed by Duncan’s multiple range tests to estimate statistical significance at the level of *p* < 0.05. Furthermore, the LC-MS data were acquired and analyzed by MassHunter Acquisition B.03.01 and Qualitative Analysis B.03.01. The MassBank (http://www.massbank.jp, accessed on 25 April 2017), ChemSpider (http://www.chemspider.com, accessed on 6 May 2017), and Phenol-Explorer (www.phenol-explorer.eu, accessed on 2 May 2017) databases were used to analyze the MS^n^ data.

## 3. Results and Discussion

### 3.1. Change in Phenolic Profiles of THR Extract during In Vitro Digestion

The changes of total phenolic and flavonoid content of THR extract during the in vitro simulated digestion were explored. In vitro digestion model has been widely used to study changes in food composition [20]. As shown in Table 1, the total phenolic and flavonoid contents of the THR extract decreased obviously after oral, stomach, and intestinal digestions. After oral digestion, the total phenolic content decreased slightly from 225.38 to 214.13 mg GAE/g DW, and the total flavonoid decreased from 124.95 to 109.47 mg CAE/g DW. This result was different from the previous reports, which showed that short-term oral digestion and α-amylase had little effect on the polyphenols, and that oral digestion can be omitted [11,14]. However, Nada Bahloul et al. [21] reported that polyphenols, the aqueous extracts from Tunisian diplotaxis, were digested in large quantities in the oral digestion compared to stomach and intestinal digestions. The discrepant results may be due to the different structures of the phenolic substances extracted by various substances. Obviously, after gastric digestion, the total phenolic content reduced significantly from 214.13 to 104.61 mg GAE/g DW and total flavonoid decreased from 109.47 to 83.64 mg CAE/g DW, which indicated that many components were unstable in the stomach environment, such as the ring cleavage of anthocyanins [22]. However, Mosele et al. [11] studied the stability and metabolism of *Arbutus unedo* bioactive compounds and found that polyphenols were stable in the stomach, which might be protected by a certain amount of pectin formed in the gel. According to our previous study [3], rutin and isoquercitrin were the main components in THR extract, which had a high rate of metastasis when passed through the small intestine wall [23,24]. Interestingly, the polyphenol and flavonoid in the THR extract appeared to be more stable in the intestine, especially flavonoids. After intestinal digestion, total phenolic content decreased from 104.61 to 97.53 mg GAE/g DW and total flavonoid content decreased slightly from 83.64 to 81.98 mg CAE/g DW. It was suggested that the phenolic components of the THR extract were degraded to some extent during the digestion of the gastrointestinal tract.

**Figure 1:**
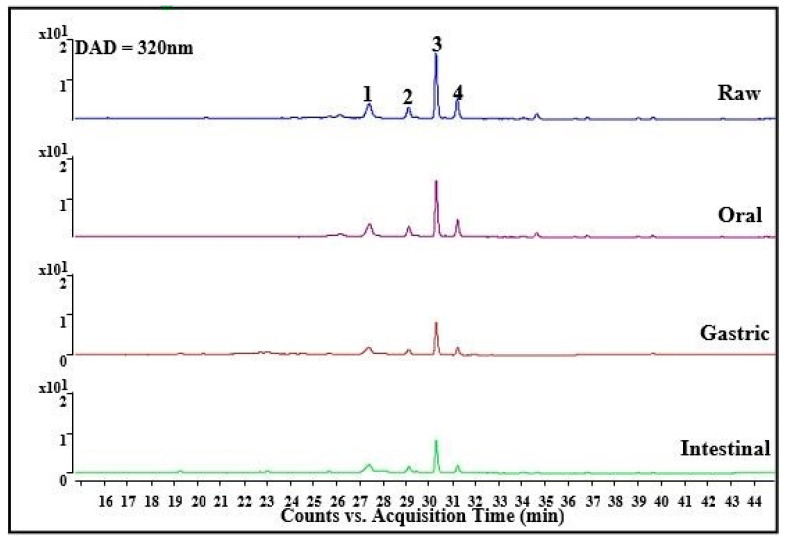
The change of the major phenolics in THR extract during the in vitro gastrointestinal digestion.

Gastrointestinal digested samples were analyzed by LC-QTOF-MS/MS and LC-QqQ-MS/MS. There are four distinct peaks in Figure 1. According to our previous report [3], quercetin-3-rutinoside (rutin, peak 1), quercetin-3-glucoside (isoquercitrin, peak 2), kaempferol-3-rutinoside (peak 3), and kaempferol-3-glucoside (peak 4) were identified. As shown in Table 2, the changes in the contents of four substances mainly occurred in the gastric digestion compared to the oral and intestinal digestions. Kaempferol-3-rutinoside was the most abundant of the four substances, as shown in Table 2, reaching 53.48 mg kaempferol/g DW. From the mouth to the intestine, the content of rutin reduced by about half, but it was more stable in the gastrointestinal tract [25]. This might have been caused by two factors. Firstly, rutin may interact with other substances (such as proteins or polysaccharides) to form a complex, which could not be detected in the present analytical method. Secondly, there may be significant differences in the in vitro digestion parameters and representation methods, such as filtration, centrifugation, membrane dialysis, etc. [25]. On the other hand, isoquercitrin can be degraded into quercetin due to digestive enzymes in the stomach [26] or degraded into aglycon and quercetin in the gut, which were absorbed or entered the colon degraded by microorganisms [27]. In addition, it has been reported that isoquercitrin can be absorbed directly into the body [23,24]. Kaempferol glycosides are also unstable under oral and gastrointestinal conditions. It has been reported that kaempferol glycosides can be hydrolyzed to aglycone kaempferol in oral and intestine digestion by glycosidases, which will bind to starch in the digestive tract [28]. During gastrointestinal digestion, part of kaempferol glycosides were degraded or absorbed, and another part entered the lower digestive tract to continue to metabolize. It has been reported that about 48% of dietary polyphenols were bioaccessible in the small intestine and 42% in the large intestine, while the rest were not accessible [29]. Because of the limitation of chromatographic conditions, the corresponding metabolites were not found.

The polyphenol content in food does not represent the amount of metabolic absorption in the body. During the digestion process, various components are continuously exposed to physical (temperature), chemical (pH), and biological (enzyme) conditions [11], which will affect the bioavailability and biological activity of potential food bioactive compounds [13]. On the other hand, food matrices should be considered, which affect the stability of polyphenols during gastric and intestinal digestion and the proportion of phenolic compounds that reach the colon or are absorbed [14]. The food we consume every day contains a lot of polyphenols, but, in fact, only a small part can be absorbed. Demethylation and deglycosylation are the main ways polyphenols are metabolized, which can improve their effectiveness of [30]. Some in vitro studies showed that the phenolic compounds can be transported through the intestinal epithelial cells in the form of glycosides via sugar transporters [22]. After absorption, they were hydrolyzed to aglycons by β-glucosidase. Moreover, aglycones can also be formed in the lumen through the action of membrane-bound lactase phlorizin hydrolase (LPH), which was absorbed passively through the epithelium, as compared with conjugation in the ileal epithelium or the liver. Hepatic metabolites (methylated, sulfated, or glucuronidated conjugates) were returned via the enterohepatic circulation (in bile) to the gut lumen [22,31].

### 3.2. Change in Phenolic Profiles of THR Extract during In Vitro Colonic Fermentation

**Figure 2:**
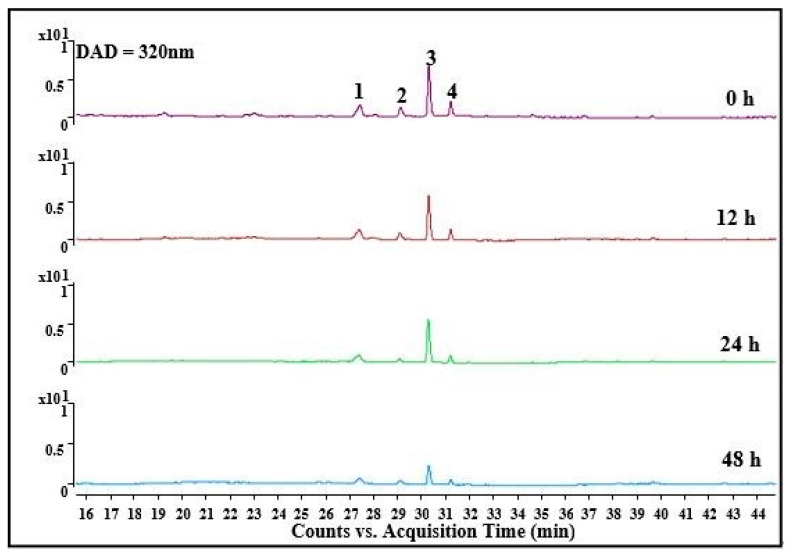
The change of the major phenolics in THR extract during colonic fermentation by human microflora.

The gastrointestinal digested samples were further used to carry out colonic fermentation because intestinal microbes are an important part of our entire digestive ecosystem. It is important to study the effects of microorganisms on the digestion and absorption of phenols. As shown in Table 1, one-third of the total phenolics and half of the flavonoids, which were not digested in the gastrointestinal tract, entered the colon. However, this result was not the same as Mosele et al. [14], who reported that most of the phenolics entered into the colon. When polyphenols reached the colon, they were absorbed intactly through the epithelium or metabolized by the colonic microbiotas [22]. Analyzed with LC-QTOF-MS/MS, four substances were found as shown in Figure 2, including quercetin-3-rutinoside (rutin, peak 1), quercetin-3-glucoside (isoquercitrin, peak 2), kaempferol-3-rutinoside (peak 3), and kaempferol-3-glucoside (peak 4). Some small peaks were also shown in Figure 2, which could not be detected with the existing chromatographic conditions due to the low content. After 48 h of fermentation, the total phenolic content reduced from 86.93 to 33.85 mg GAE/g DW and the total flavonoid content reduced from 72.44 to 17.56 mg CAE/g DW, indicating that colonic microorganisms can decompose these polyphenols. As Mosele et al. [14] reported, tannic acid and ellagic acid were metabolized into urolithins by microorganisms, which were considered to be a potentially biologically active microbial metabolite.

As shown in Table 2, it was obvious that the contents of the four substances dropped to a lower level after 48 h of microbial metabolism. After 24 h of fermentation, the contents of quercetin-3-rutinoside and quercetin-3-glucoside remained basically unchanged. However, kaempferol-3-rutinoside and kaempferol-3-glucoside were degraded throughout the fermentation process. It has been proven that rutin was degraded into quercetin and further converted into 3-hydroxylphenylacetic acid under the action of microorganisms in the colon [20]. In addition, rutin can be converted to isoquercitrin by microorganisms under anaerobic condition [32], and isoquercitrin was decomposed into quercetin and glucose by microorganisms through O-deglycosylation [33]. Kaempferol glycosides were reported to be degraded to kaempferol and corresponding sugar ligands through deglycosylation. Kaempferol was further degraded to form 3-(4-hydroxyphenyl) propionic acid through ring fission under the action of microorganisms, which was further degraded to form 3-phenylpropionic acid and phenylacetic acid. 3-Phenylpropionic acid can also be converted to phenylacetic acid [34]. Many substances, which are digested difficultly in the gastrointestinal tract, can be degraded under the action of colonic microorganisms. Chlorogenic acid is considered to be a substance that is difficult to be absorbed in the gastrointestinal tract. It has even been reported to be resistant to intestinal enzymes [35] but can be absorbed after being broken down into small molecules by microorganisms [36]. Chlorogenic acid was one of the main components of THR extract in our previous identification [3]. It can be degraded into caffeic acid and quinic acid by microorganisms, and caffeic acid can be converted into ferulic acid by deacetylation [20]. 3-(3-Hydroxyphenyl) propionic acid, a decomposition product of caffeic acid, was metabolized through the transport of Caco-2 cells in liver [37]. In the metabolic absorption of the colon, phenolic compounds entering the colon were mainly composed of unabsorbed glycosides and conjugates that pass through the ileal and hepatic metabolic cycles [22,31]. Flavonoids that entered the colon were metabolized into simple phenolic acid under the action of microorganisms with the form of fission, ring-opening, and other degradation [22], which entered the circulation via transporters or by passive diffusion [37].

### 3.3. In Vivo Antioxidant Activities of THR

**Figure 3:**
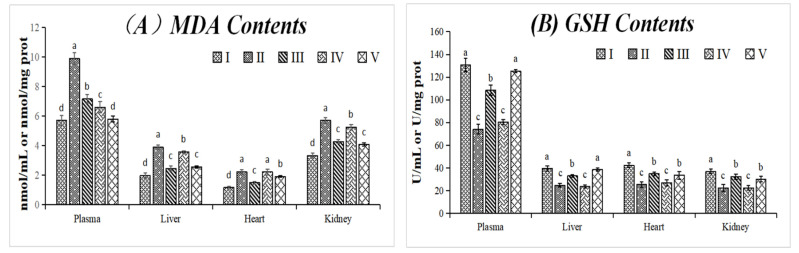

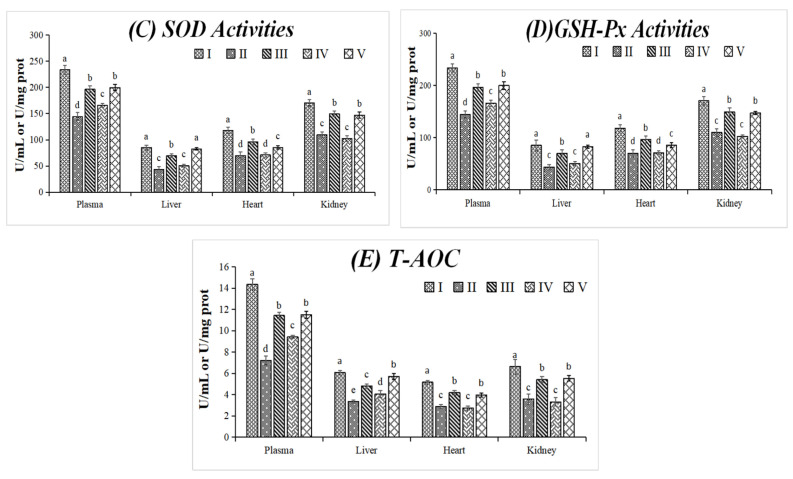
The changes of (**A**) MDA contents; (**B**) GSH contents; (**C**) SOD activities; (**D**) GSH-Px activities; and (**E**) T-AOC in different groups. Data were presented as mean ± SD (*n* = 8); values not sharing a common superscript letter denote significant difference (*p* < 0.05). Rats were divided into 5 groups as follows: I: normal control group (saline); II: model control group (D-gal solution, 200 mg/kg BW); III: positive control group (VC solution, 200 mg/kg BW), IV: low dosage group (THR extract, 200 mg/kg BW), and V: high dosage group (THR extract, 1000 mg/kg BW). Please note that the data of the I, II, and III groups were the same as we used in our previous paper (*Journal of Functional Foods* 2017, 30, 179–193), experiments for which were carried out simultaneously with the current paper.

The MDA and GSH contents and the antioxidant capacity of SOD, GSH-Px, and T-AOC enzymes in plasma, liver, heart, and kidney samples of rats under different treatment regimens were tested to evaluate the antioxidant activity of THR extract. The effects of THR extract were also compared with the positive control of VC. Results are shown in Figure 3.

#### 3.3.1. Effect on MDA and GSH Contents

MDA and GSH contents can reflect the level of oxidative stress in the body. As shown in Figure 3A,B, compared with the normal control group (group I), the MDA content increased significantly and GSH content reduced in plasma and various tissues in the model control group (group II), which showed that the D-gal model was established successfully. As shown in Figure 3A, MDA contents were reduced significantly, or even lower than the positive control group in plasma, in both low- and high-dosage groups. It indicated that THR extract can effectively reduce the lipid peroxidation. As shown in Figure 3B, compared with the model control group (group I), the GSH contents remained basically unchanged in the low dosage group (group IV), but increased significantly in the high-dosage group (group V). It was indicated that the effect of the THR extract was dose-dependent. A high-dose (1000 mg/kg BW) THR extract had a significant effect on the reduction of MDA and the increase of GSH, which was similar to or stronger than the positive control (group III, 200 mg VC/kg BW). Interestingly, the contents of MDA and GSH were the highest in plasma compared to other tissues. THR extract showed significant beneficial effects, especially at higher doses, that ultimately contributed to reducing oxidative stress and protected rats from oxidative damage in different tissues and organs.

#### 3.3.2. Effects on SOD, GSH-Px, and T-AOC Activities

SOD and GSH-Px are important antioxidant enzymes in the body. As shown in Figure 3C–E, the SOD, GSH-Px, and T-AOC activities were reduced significantly by D-gal solution (group II) compared with the blank control group (group I), which showed that the D-gal model was established successfully. From group IV and group V, it was clear that there was a dose-dependent effect on SOD, GSH-Px and T-AOC activities. Low-dose THR extract (group IV) showed significant effects on SOD, GSH-Px, and T-AOC activities in plasma, but no significant effects were found in other tissues except the T-AOC in the liver. However, the SOD, GSH-Px, and T-AOC activities were improved obviously in plasma and other tissues treated with high-dose THR extract. The activity of SOD and GSH-Px in the plasma and kidney were higher than those in the liver and heart (Figure 3C,D). However, the T-AOC activity was higher in the plasma than in other tissues (Figure 3). High-dose THR extract (1000 mg/kg BW) showed significantly stronger T-AOC activity than VC (Figure 3E). In general, the use of THR extract can increase the antioxidant capacity of rats and reduce the damage caused by D-gal.

#### 3.3.3. The Relationship between Metabolites and In Vivo Antioxidant Activities

It was shown that gastrointestinal digestion and colonic fermentation had significant effects on the antioxidant activity of polyphenol. The structure of polyphenols was changed in these degradation processes, leading to the change of antioxidant activity [20]. Luzardo-Ocampo et al. [24] found that the antioxidant activity of digested corn (*Zea mays* L.) and common beans (*Phaseolus vulgaris* L.) were higher than their methanol extracts, but Bao et al. [38] found that the antioxidant activity of flavonoids from tartary buckwheat rice decreased after in vitro digestion. Food matrix showed a significant impact on digestion; generally, free polyphenols were extracted but there are many bound polyphenols in food that may be released during digestion. Of course, the antioxidant activity changed due to the polyphenol structural modification when the extracts were used for in vitro digestion. It has been reported that the antioxidant activity of the parent phenols was reduced under microbial action, but the overall antioxidant capacity did not diminish due to the accumulation of metabolites that might produce the same antioxidant capacity as the parents [20]. However, the antioxidant activity of metabolites was higher than that of their parents in some cases, such as dihydroferulic acid, a metabolite of chlorogenic acid [20]. Although in vitro antioxidant experiments cannot reflect in vivo antioxidants, they can be used as a reference.

It has been reported that bioactive components in diets act in two ways: by directly scavenging free radicals and/or by activating the transcription of cytoprotective enzymes involved in the detoxification of xenobiotics [22]. On one hand, polyphenols and their metabolites play an antioxidant role directly, which can be absorbed directly or after being metabolized in gastrointestinal digestion and colon fermentation. On the other hand, polyphenols and some simple phenolic acids as their metabolites can activate the Keap1/Nrf2/ARE (Kel-Ch ECH associating protein 1/nf-e2-related factor 2/Antioxidant The Response Elements) pathway, and these active substances can modify the key cysteine residues on Keap1, which enables binding to NRF-2 and migration of the activated complex into the nucleus, where it activates genes with antioxidant elements, coding antioxidant enzymes such as superoxide dismutase, catalase, glutathione synthetase, etc. [22]. Rats treated with THR extract showed positive effects on MDA, GSH, SOD, GSH-Px, and T-AOC (Figure 3). The dose-dependence indicated that polyphenols in THR have good bioavailability, and the dietary intake of foods with higher polyphenols has potential benefits for oxidative stress. Therefore, THR as a traditional functional ingredient with good biological value has great potential.

## 4. Conclusions

The stability and catabolism of THR were evaluated under in vitro simulated gastrointestinal digestion and colonic fermentation models. Total phenolic and flavonoid content were degraded during each digestion process. THR extract showed higher gastrointestinal digestibility and less reaching the colon; in other words, it may be absorbed in the gastrointestinal tract and have high bioavailability. Quercetin-3-rutinoside, quercetin-3-glucoside, kaempferol-3-rutinoside, and kaempferol-3-glucoside were the main substances of THR, which were degraded at various stages, while their in vivo metabolites [3] could help explain the favorable changes in several antioxidant biomarkers (SOD, GSH-Px, T-AOC) and the lipid peroxidation product (MDA) in rats treated with THR extract. This also proved that THR is reasonable as a traditional functional food and is effective to treat chronic diseases caused by oxidative stress.

## Figures and Tables

**Table 1:** Total phenolic content and total flavonoid content of THR extract during in vitro gastrointestinal digestion and colonic fermentation by human microflora ^A^.

Sample	Total Phenolic Content (mg GAE/g DW) ^B^	Total Flavonoid Content (mg CAE/g DW) ^C^
Raw	225.38 ± 2.62 ^h^	124.95 ± 3.31 ^g^
In vitro gastrointestinal digestion		
Oral digestion	214.13 ± 2.34 ^g^	109.47 ± 1.78 ^f^
Gastric digestion	104.61 ± 1.51 ^f^	83.64 ± 2.14 ^e^
Intestinal digestion	97.53 ± 2.47 ^e^	81.98 ± 1.37 ^e^
In vitro colonic fermentation by human microflora		
0 h	86.93 ± 2.09 ^d^	72.44 ± 1.56 ^d^
12 h	78.27 ± 1.88 ^c^	61.25 ± 1.08 ^c^
24 h	65.42 ± 3.53 ^b^	49.84 ± 1.67 ^b^
48 h	33.85 ± 2.05 ^a^	17.56 ± 1.29 ^a^

^A^ Results are expressed as mean ± standard deviation of three replicates. Values followed by different letters (a–h) within the same column are significantly different (*p* < 0.05). ^B^ Phenolic contents are expressed as mg gallic acid equivalents (GAE) in 1 g of dry weight of extracts ± standard deviation. ^C^ Flavonoid contents are expressed as mg catechin equivalents (CAE) in 1 g of dry weight of extracts ± standard deviation.

**Table 2:** Identification and quantification of the major phenolics in THR extract during the in vitro gastrointestinal digestion and colonic fermentation by human microflora ^A^.

No.	Compounds ^B^	t_R_ (min)	Parent/Daughter Ions (*m*/*z*)	Raw	Gastrointestinal Digestion			Colonic Fermentation by Human Microflora			
					Oral	Gastric	Intestinal	0 h	12 h	24 h	48 h
1	Quercetin-3-rutinoside	27.41	609/301	16.86 ± 1.19 ^h^	14.09 ± 1.25 ^g^	8.77 ± 0.86 ^f^	8.04 ± 0.45 ^d^	8.12 ± 0.79 ^e^	6.70 ± 0.26 ^c^	3.37 ± 0.03 ^b^	3.19 ± 0.40 ^a^
2	Quercetin-3-glucoside	29.18	463/301	12.55 ± 1.03 ^h^	11.24 ± 1.31 ^g^	7.18 ± 1.12 ^f^	6.63 ± 0.58 ^e^	6.57 ± 0.58 ^d^	4.92 ± 0.68 ^c^	2.48 ± 0.11 ^b^	2.42 ± 0.21 ^a^
3	Kaempferol-3-rutinoside	30.39	593/285	53.48 ± 3.47 ^h^	49.13 ± 2.76 ^g^	27.94 ± 1.34 ^f^	27.88 ± 1.07 ^e^	24.5 ± 2.01 ^d^	21.98 ± 1.91 ^c^	21.46 ± 1.31 ^b^	7.67 ± 0.55 ^a^
4	Kaempferol-3-glucoside	31.26	447/285	19.97 ± 1.57 ^h^	16.40 ± 1.44 ^g^	9.54 ± 0.95 ^e^	9.73 ± 1.04 ^f^	9.38 ± 0.82 ^d^	7.60 ± 0.64 ^c^	4.54 ± 0.08 ^b^	2.88 ± 0.09 ^a^

^A^ Results are expressed as mean ± standard deviation of three replicates. Values followed by different letters (a–h) within the same line are significantly different (*p* < 0.05). ^B^ Peaks 1 and 2 were expressed as quercetin equivalents per g dry weight extract (mg quercetin/g DW). Peaks 3 and 4 were expressed as kaempferol equivalents per g dry weight extract (mg kaempferol/g DW).
